# Supplementary material for: Association of trait and specific hopes: cross sectional study on students and workers of health professions in Split, Croatia
Source: PeerJ. 2016 Jan 19;4:e1604. doi: 10.7717/peerj.1604 (PMC4727959; doi:10.7717/peerj.1604)
Supplement: Supplemental Information 1 [file peerj-04-1604-s001.docx]

### Supplementary Table 1. Integrative hope scale (IHS) scores and levels of hope for specific events for students (n=84) who first filled out IHS questionnaire and those (n=77) who first filled out the visual analogue scales (VAS).

| **Variable** | **Integrative hope scale scores (Md, IQR) of students who first filled** | | ***P**** |
| --- | --- | --- | --- |
|  | **CIHS** | **VAS** |  |
| **Integrative hope total score** | 108.0 (104-118) | 112.0 (108-119) | 0.07 |
| **Integrative hope subscale** |  |  |  |
| trust and confidence | 33.0 (30-36) | 34.0 (32-36) | 0.20 |
| lack of perspective | 26.5 (23-30) | 27.5 (24-31) | 0.06 |
| positive future orientation | 21.0 (20-23) | 22.0 (20-33) | 0.32 |
| social relations and personal value | 24.0 (22-26) | 24.0 (23-26) | 0.46 |
| **Levels of hope** |  | | |
| for finishing studies in time | 90 (80-100) | 90 (80-100) | 0.59 |
| for being healthy at the age of 60 | 70 (60-80) | 70 (60-80) | 0.95 |
| for the thing most hoped-for in life | 90 (75-100) | 90 (80-100) | 0.95 |

* Mann-Whitney U test.

**Supplementary Table 2. Agreement of factor rang order among students (n=161) and workers (n=88) of health professions.**

| **Agreement (W*)** | **Hope for finishing studies in time** | **Hope for being healthy at the age of 60** | **Most hoped-for thing in life†** | **Trait hope factors** |
| --- | --- | --- | --- | --- |
| students | 0.024 | 0.053 | 0.027 | 0.384 |
| workers | / | 0.158 | 0.117 | 0.304 |
| students and workers | / | 0.072 | 0.035 | 0.338 |
| students with high trait hope ‡ | 0.007 | 0.121 | 0.017 | / |
| students with low trait hope§ | 0.130 | 0.051 | 0.004 | / |
| workers with high trait hope‡ | / | 0.146 | 0.069 | / |
| workers with low trait hope§ | / | 0.173 | 0.230 | / |

***** Kendall's coefficient of concordance.

† Chi-square test.

‡1^st^ quartile of trait hope total scores measured by Integrative hope scale

§4^th^ quartile of trait hope total scores measured by Integrative hope scale

**Supplementary table 3. Factors which students (n=161) and workers (n=88) of health professions selected as most contributing to their level of hope for specific events.**

| **Hope type** | **Most contributing factor** | | | | **P*** |
| --- | --- | --- | --- | --- | --- |
|  | **optimism** | **ambition** | **social support** | **self-confidence** |  |
| for finishing studies in time |  |  |  |  |  |
| Students (%) | 23.7 | 32.3 | 25.3 | 18.7 | 0.075 |
| for being healthy at the age of 60 |  |  |  |  |  |
| Students (%) | 41.2 | 20.0 | 24.1 | 14.7 | <0.001 |
| Workers (%) | 46.9 | 13.5 | 20.8 | 18.8 | <0.001 |
| the most hoped-for thing in life |  |  |  |  |  |
| Students (%) | 35.4 | 22.7 | 25.4 | 16.5 | 0.004 |
| Workers (%) | 44.7 | 13.6 | 22.3 | 19.4 | <0.001 |
| Trait hope† |  |  |  |  |  |
| Students (%) | 9.1 | 69.9 | 17.6 | 3.4 | <0.001 |
| Workers (%) | 6.4 | 45.5 | 34.5 | 13.6 | <0.001 |

* χ^2^ test.

†Measured by Integrative hope scale.

**Appendix 1.**

All supplementary materials are freely available and attached to the manuscript. All anonymized data is freely available on request. For permission to use Integrative hope scale in your own research please contact Beate Schrank, e-mail: [beate.schrank@meduniwien.ac.at](mailto:beate.schrank@meduniwien.ac.at).

Integrative hope scale – Croatian Version (item translation):

1. Imam jaku unutarnju snagu.

2. Teško mi je održati interes za aktivnosti u kojima sam prije uživao.

3. Postoje stvari koje želim postići u životu.

4. Osjećam se voljeno.

5. I kada drugi posustanu, ja znam da mogu naći rješenje problema.

6. Čini se kao da mi je sva potpora uskraćena.

7. Osjećam kojim putem trebam ići.

8. Unaprijed se veselim aktivnostima koje volim.

9. Vjerujem da svaki dan nudi nove mogućnosti.

10. Muče me nevolje koje sprječavaju moje planiranje budućnosti.

11. Imam nekoga tko dijeli moje nedoumice.

12. Ja vidim mogućnosti i u moru poteškoća.

13. Nemam nade za neke dijelove moga života.

14. Potreban sam drugima.

15. Osjećam da moj život ima vrijednost i značaj.

16. Osjećam se zarobljeno, okovano.

17. Ja planiram svoju budućnost.

18. Dosad sam bio poprilično uspješan u životu.

19. Primjećujem da postajem nezainteresiran za većinu stvari u životu.

20. Cijenjen sam po onome što sam.

21. Moja prijašnja iskustva su me dobro pripremila za moju budućnost.

22. Namjeravam živjeti punim plućima.

23. Imam vjeru i ona mi pruža utjehu.

On the following line please mark how much you hope to finish your studies in time (within 3 years):

highest

no hope possible hope

|  |  |  |  |  |  |  |  |  |  |  |
| --- | --- | --- | --- | --- | --- | --- | --- | --- | --- | --- |
|  |  |  |  |  |  |  |  |  |  |  |

0 10 20 30 40 50 60 70 80 90 100

Please rank each of the following factors with numbers 1 to 4 according to how much they contributed to the strength (level) of your hope from the previous question (1 being most, 4 – being least; use each number/value only once).

my optimism ____ my ambition____

my self-confidence____ support of my surroundings____

On the following line please mark how much you hope to be healthy at the age of 60:

highest

no hope possible hope

|  |  |  |  |  |  |  |  |  |  |  |
| --- | --- | --- | --- | --- | --- | --- | --- | --- | --- | --- |
|  |  |  |  |  |  |  |  |  |  |  |

0 10 20 30 40 50 60 70 80 90 100

Please rank each of the following factors with numbers 1 to 4 according to how much they contributed to the strength (level) of your hope from the previous question (1 being most, 4 – being least; use each number/value only once).

my optimism ____ my ambition____

my self-confidence____ support of my surroundings____

What do you hope for most in life? _____________________________________

What strength would you attach to that hope (hoping):

highest

no hope possible hope

|  |  |  |  |  |  |  |  |  |  |  |
| --- | --- | --- | --- | --- | --- | --- | --- | --- | --- | --- |
|  |  |  |  |  |  |  |  |  |  |  |

0 10 20 30 40 50 60 70 80 90 100

Please rank each of the following factors with numbers 1 to 4 according to how much they contributed to the strength (level) of your hope from the previous question (1 being most, 4 – being least; use each number/value only once).

my optimism ____ my ambition____

my self-confidence____ support of my surroundings____
